# Characterization of CRISPR-Cas Systems in Clinical *Klebsiella pneumoniae* Isolates Uncovers Its Potential Association With Antibiotic Susceptibility

**DOI:** 10.3389/fmicb.2018.01595

**Published:** 2018-07-16

**Authors:** Hsin-Yu Li, Cheng-Yen Kao, Wei-Hung Lin, Po-Xing Zheng, Jing-Jou Yan, Ming-Cheng Wang, Ching-Hao Teng, Chin-Chung Tseng, Jiunn-Jong Wu

**Affiliations:** ^1^Department of Biotechnology and Laboratory Science in Medicine, School of Biomedical Science and Engineering, National Yang Ming University, Taipei, Taiwan; ^2^Department of Internal Medicine, National Cheng Kung University Hospital, College of Medicine, National Cheng Kung University, Tainan, Taiwan; ^3^Biotechnology Center in Southern Taiwan, Agricultural Biotechnology Research Center, Academia Sinica, Tainan, Taiwan; ^4^Department of Pathology, Cheng Ching Hospital at Chung Kang, Taichung, Taiwan; ^5^Institute of Molecular Medicine, College of Medicine, National Cheng Kung University, Tainan, Taiwan

**Keywords:** CRISPR-Cas systems, *Klebsiella pneumoniae*, urinary tract infection, bacteremia, antibiotic susceptibility

## Abstract

Prokaryotic CRISPR-Cas systems limit the acquisition of genetic elements and provide immunity against invasive bacteriophage. The characteristics of CRISPR-Cas systems in clinical *Klebsiella pneumoniae* isolates are still unknown. Here, 97 *K. pneumoniae* genomes retrieved from the Integrated Microbial Genomes & Microbiomes genome database and 176 clinical isolates obtained from patients with bloodstream (BSI, *n* = 87) or urinary tract infections (UTI, *n* = 89) in Taiwan, were used for analysis. Forty out of ninety-seven genomes (41.2%) had CRISPR-Cas systems identified by the combination of CRISPRFinder and *cas1* gene sequence alignment. The phylogenetic trees revealed that CRISPR-Cas systems in *K. pneumoniae* were divided into two types (type I-E, 23; subtype I-E^∗^, 17) based on the sequences of Cas1 and Cas3 proteins and their location in the chromosome. The distribution of type I-E and I-E^∗^ CRISPR-Cas systems was associated with the multilocus sequence typing and the pulsed-field gel electrophoresis results. Importantly, no CRISPR-Cas system was identified in published genomes of clonal complex 258 isolates (ST11 and ST258), which comprise the largest multi-drug resistant *K. pneumoniae* clonal group worldwide. PCR with *cas*-specific primers showed that 30.7% (54/176) of the clinical isolates had a CRISPR-Cas system. Among clinical isolates, more type I-E CRISPR-Cas systems were found in UTI isolates (BSI, 5.7%; UTI, 11.2%), and subtype I-E^∗^ CRISPR-Cas systems were dominant in BSI isolates (BSI, 28.7%; UTI, 15.7%) (*p* = 0.042). Isolates which had subtype I-E^∗^ CRISPR-Cas system were more susceptible to ampicillin-sulbactam (*p* = 0.009), cefazolin (*p* = 0.016), cefuroxime (*p* = 0.039), and gentamicin (*p* = 0.012), compared to the CRISPR-negative isolates. The strains containing subtype I-E^∗^ CRISPR-Cas systems had decreased numbers of plasmids, prophage regions, and acquired antibiotic resistance genes in their published genomes. Here, we first revealed subtype I-E^∗^ CRISPR-Cas system in *K. pneumoniae* potentially interfering with the acquisition of phages and plasmids harboring antibiotic resistance determinants, and thus maintained these isolates susceptible to antibiotics.

## Introduction

During bacterial evolution, the ability of bacteria to adapt to various hosts and environments has been favored by the acquisition of DNA elements through horizontal gene transfer (HGT) (Frost et al., 2005; Ilyas et al., 2017). An adaptive immune system, clustered regularly interspaced short palindromic repeats and their associated Cas proteins (CRISPR-Cas), which has been identified in many species of bacteria, allows bacteria to limit the acquisition of external genetic elements, and thus balances the need to acquire beneficial characteristics through HGT with the need to defend against infection by bacteriophage (Barrangou, 2015; Jiang et al., 2016; Hoyland-Kroghsbo et al., 2017). Moreover, the CRISPR-Cas systems have been shown to play a critical role in the exchange of genetic elements, biofilm formation, colonization, and virulence regulation in multiple pathogenic bacteria (Shah and Garrett, 2011; Louwen et al., 2013, 2014; Almendros et al., 2014).

The CRISPR elements are composed of short direct repeat sequences (DR) separated by unique sequences (spacers) that range in size from 26 to 72 bp and originate from mobile genetic elements, such as phages, transposons, or plasmids (Barrangou, 2015; Koonin et al., 2017). Based on the *cas* gene content, *cas* operon architecture, Cas protein sequences, and processes that underlie the aforementioned steps, CRISPR-Cas systems are divided into two main classes, which encompass 6 major types and 33 different subtypes (Makarova and Koonin, 2015; Makarova et al., 2015; Koonin et al., 2017). The number of *cas* genes varies from 4 to over 20, and the Cas proteins contain a variety of enzymatic domains with nuclease, helicase, or polymerase activity (Makarova et al., 2011; Koonin et al., 2017). Therefore, Cas proteins with enzymatic activities are required for the acquisition of spacers as well as for marking the invader elements (Nunez et al., 2014).

*Klebsiella pneumoniae* is a Gram-negative bacteria belonging to the family Enterobacteriaceae, closely related to the pathogens *Salmonella enterica* and *Escherichia coli*. *K. pneumoniae* has been the most frequent pathogen causing hospital-acquired infections and has high rates of antibiotic resistance (Gomez-Simmonds and Uhlemann, 2017). The multi-drug resistance (MDR) and hypervirulent phenotype of *K. pneumoniae* are commonly associated with the presence of high molecular weight plasmids (Mathers et al., 2015; Lee et al., 2017). CRISPR-Cas systems are shown to be associated with antibiotic susceptibility in *Streptococcus pyogenes* and *E. coli* (Zheng et al., 2014; Aydin et al., 2017). The CRISPRI-F system is found potentially interfering with the acquisition of antibiotic-resistant plasmids, and thus CRISPRI-F system is dominant in antibiotic-susceptible *E. coli* (Aydin et al., 2017). However, the association of CRISPR-Cas systems and antibiotic resistance in *K. pneumoniae* is still unclear.

The prevalence of CRISPR-Cas systems among *Klebsiella* genomes was very different using CRISPERFinder analysis (Ostria-Hernandez et al., 2015; Shen et al., 2017). Ostria-Hernandez et al. (2015) reported that CRISPR-Cas systems were identified in 6 out of the 52 (11.5%) *K. pneumoniae* genome sequences by using CRISPRFinder. In contrast, a total of 37 (54.4%) CRISPR-Cas systems were found by CRISPERFinder among the 68 *Klebsiella* genomes retrieved from the NCBI genome database (Shen et al., 2017). Moreover, three different CRISPR-Cas systems (type I-E, type I-F, and subtype I-E^∗^) were found in the published *Klebsiella* genomes (Shen et al., 2017). Type I-E and subtype I-E^∗^ CRISPR-Cas systems were found in *K. pneumoniae* and *K. oxytoca*, and type I-F CRISPR-Cas system was identified in only *K. oxytoca* (Shen et al., 2017). In this study, we characterized the CRISPR-Cas systems in *K. pneumoniae* published genomes and clinical isolates obtained from patients with bloodstream infection (BSI) or urinary tract infection (UTI) in Taiwan and found that isolates having subtype I-E^∗^ CRISPR-Cas system are more susceptible to certain antibiotics.

## Materials and Methods

### CRISPR-Cas System Identification

A total of 97 complete genome sequences of *K. pneumoniae* (including chromosome and plasmids sequences), were retrieved from the Integrated Microbial Genomes & Microbiomes (IMG/M) genome database^1^. CRISPRFinder was used to identify the presence of CRISPR-Cas systems and spacers among the genomes^2^. Given that Cas1 is considered a genetic and universal marker for the CRISPR-Cas systems, BLASTn of *cas1* and *cas3* genes was used to validate the results of CRISPRFinder^3^. Sequence types (STs) were determined based on a comparative analysis of seven housekeeping genes (*rpoB*, *gapA*, *mdh*, *pgi*, *phoE*, *infB*, and *tonB*) against the *K. pneumoniae* PubMLST database^4^. The software MEGA X was used to perform a Maximum Likelihood-based phylogenetic tree (Jukes–Cantor model) based on aligned nucleotide sequences of concatenated multilocus sequence typing (MLST) genes (12,096 bp) of CRISPR-Cas positive *K. pneumoniae* genomes (Kumar et al., 2018).

PHAge Search Tool (PHAST) was used to identify, annotate, and graphically display prophage sequences within *K. pneumoniae* genomes^5^ (Zhou et al., 2011). Acquired antibiotic resistance genes carried on plasmids, including aminoglycoside, β-lactam, fluoroquinolones, MLS (macrolides, lincosamides, and streptogramines), rifampicin, phenicols, sulfonamide, tetracycline, and trimethoprim resistance genes, were identified by ResFinder 3.0 using BLAST for identification of acquired antibiotic resistance genes in plasmid sequences of the 97 genomes^6^ (Zankari et al., 2012).

### Sampling and Isolation of *K. pneumoniae*

Eighty-seven and eighty-nine non-consecutive, non-duplicate clinical isolates were obtained from patients with BSI and UTI respectively, at the National Cheng Kung University Hospital, Taiwan. These isolates were randomly collected and excluded the outbreak strains over a period of 6 years, between 2011 and 2016. *K. pneumoniae* isolates were identified by colony morphology, Gram stain, biochemical tests, and the Vitek 2 system (bioMérieux, Marcy-l’Etoile, France) according to the manufacturer’s recommendations. *K. pneumoniae* was grown on Luria-Bertani (LB) agar or broth. The isolates were stored at -80°C in LB broth containing 20% glycerol (v/v) until used.

### DNA Techniques

Primers used in this study are listed in Supplementary Table S1. DNA was extracted with the boiling method. In brief, 5 mL bacterial culture was grown for 16 h, and was centrifuged (3,500 rpm, 5 min). The pellet was resuspended in 1.0 mL of TE buffer (EDTA 1.0 mM; Tris-HCl 10 mM, pH = 8.0), boiled for 15 min, and was centrifuged (8,000 rpm, 5 min). The supernatant containing genomic DNA was collected and stored at 4°C until used. The PCR reactions were carried out in a total volume of 25 μL containing 1x PCR buffer, 1.5 mM MgCl_2_, 0.2 mM dNTPs, 1 unit of GoTaqDNA polymerases (Promega, Madison, WI, United States), 10 pmol of each primer and 1 μL DNA template. The PCR amplifications were performed using an ABI 2720 Thermal Cycler (Applied Biosystems, Foster City, CA, United States). The PCR conditions for*cas1* and *cas3* genes were: 95°C for 5 min, followed by 30 repeated cycles of 30 s at 95°C, 30 s of annealing at 60°C and 1 min of extension at 72°C, followed by 7 min as final extension at 72°C.

### Multilocus Sequence Typing (MLST)

Amplification of seven housekeeping genes, *rpoB*, *gapA*, *mdh*, *pgi*, *phoE*, *infB*, and *tonB*, was accomplished using PCR. Primer sets for gene amplification and sequencing were described previously (Diancourt et al., 2005), and the annealing temperatures of each primer were described at the *K. pneumoniae* PubMLST database. However, primers for *pgi* amplification showed poor specificity in this study, therefore, primers pgi-1 (GGCCGTTAGTAGAGCTGTCG) and pgi-2 (GAAGAACGTGAATCCGGAAA) were designed and used for 1,040 bp *pgi* fragment amplification and sequencing (annealing temperature: 60°C). STs were determined by comparing the nucleotide sequences against the *K. pneumoniae* PubMLST database.

### Pulsed-Field Gel Electrophoresis (PFGE)

Pulsed-field gel electrophoresis (PFGE) of *Xba*I-digested genomic DNA was carried out with a CHEF Mapper XA apparatus (Bio-Rad Laboratories, Inc., Hercules, CA, United States) using the following parameters: separation on a 1% agarose gel (Seakem Gold agarose; FMC Bio Products) in 0.5× Tris-Borate-EDTA for 19 h at 14°C with pulsed times ranging from 5 to 35 s at 6 V/cm. The gels were stained with ethidium bromide and photographed with UV transillumination. PFGE profiles were analyzed and compared using the GelCompar II software, version 2.0 (Unimed Healthcare, Inc., Houston, TX, United States).

### Antibiotic Susceptibility Testing

Antibiotic susceptibilities were determined by the disk diffusion method on Mueller-Hinton agar according to the Clinical and Laboratory Standards Institute (CLSI) guidelines (Clinical and Laboratory Standards Institute [CLSI], 2009). All clinical isolates were tested for the susceptibilities of nine antibiotics, including amikacin (30 μg/mL), ampicillin-sulbactam (10/10 μg/mL), cefotaxime (30 μg/mL), cefazolin (30 μg/mL), cefuroxime (30 μg/mL), ertapenem (10 μg/mL), gentamicin (10 μg/mL), levofloxacin (5 μg/mL), and sulfamethoxazole-trimethoprim (23.75/1.25 μg/mL) (BD BioSciences, Cockeysville, MD, United States). *E. coli* ATCC 25922 was used as quality control strain. Antibiotic susceptibility was interpreted according to the CLSI guidelines (Clinical and Laboratory Standards Institute [CLSI], 2016).

### Statistical Analysis

The Student’s *t-*test and paired *t*-tests were applied as appropriate for the parametric differences. Chi-square tests were used to test the association of a set of counts or frequencies. All tests with a *p-*value < 0.05 were taken as significant.

## Results and Discussion

### CRISPR-Cas Systems in *K. pneumoniae* Published Genomes

Forty-three out of the ninety-seven genomes (44.3%) had confirmed CRISPR loci determined by CRISPERFinder. However, no *cas1* gene was found in strains YH43, KPNIH29, DHQP1002001, and Kpn555. Therefore, these four strains were defined as CRISPR-Cas system negative in the following analysis. In contrast, strain SB3432, which had a questionable CRISPR-Cas system as determined by CRISPERFinder, was defined as CRISPR-Cas system positive strain with the Cas proteins present in the genome. Therefore, a total of 40 (41.2%) CRISPR-Cas system positive genomes were found in this study (**Table 1**).

**Table 1:** The association of CRISPR-Cas systems with chromosome size, plasmid count, phage count, spacer numbers, number of spacer hit plasmids, and the presence of antibiotic resistance genes among 97 *K. pneumoniae* genomes.

	CRISPR-Cas systems			
	Presence (*n* = 40)		Absence (*n* = 57)	*p*-Value
	Type I-E	Subtype I-E^∗^		
	(*n* = 23)	(*n* = 17)		
Chromosome size (bp)	5,354,756 (62,628)	5,339,573 (75,216)	5,346,821 (116,544)	0.761, 0.810, 0.507^a^
Plasmid count	3.52 (2.020)	1.71 (1.448)	3.02 (1.758)	0.269, **0.007**, **0.003**^a^
Phage count	5.39 (2.888)	3.18 (1.741)	6.11 (3.063)	0.341, **<0.001**, **0.008**^a^
Spacer number	36.5 (11.7)	21.3 (4.6)	–	**<0.001**^b^
Number of spacer hit plasmids	3.09 (1.6)	3.65 (1.9)	–	0.318
Presence of antibiotic resistance genes^c^				
Aminoglycoside	18 (78.3)	8 (47.1)	44 (77.2)	**0.039**^d^
β-Lactam	15 (65.2)	8 (47.1)	43 (75.4)	0.084^d^
Fluoroquinolones	17 (73.9)	7 (35.3)	36 (63.2)	0.103^d^
MLS	2 (8.7)	6 (32.3)	23 (40.4)	**0.022**^d^
Rifampicin	2 (8.7)	2 (11.8)	4 (7.0)	0.816^d^
Phenicols	5 (21.7)	12 (70.6)	29 (50.9)	0.260^d^
Sulfonamide	14 (60.9)	7 (35.3)	36 (63.2)	0.264^d^
Tetracycline	10 (43.5)	3 (17.6)	15 (26.3)	0.164^d^
Trimethoprim	14 (60.9)	6 (32.3)	32 (56.1)	0.231^d^

*^a^p-Values were shown as CRISPR-negative strains vs. type I-E strains, CRISPR-negative strains vs. subtype I-E^∗^ strains, and type I-E strains vs. subtype I-E^∗^ strains.*

*^b^p-Value was shown as type I-E strains vs. subtype I-E^∗^ strains.*

*^c^Only antibiotic resistance genes presented in the plasmid were considered (acquired resistance).*

*^d^p-Value was determined by the distribution of antibiotic resistance genes among three groups.*

*Values were expressed as mean (SD), unless otherwise indicated as number (percent).*

*MLS, Macrolides, lincosamides, and streptogramines.*

*The Student’s t-test was applied for all the parametric difference between three groups, and Pearson’s chi-square test was used for the difference in the presence of antibiotic resistance genes between three groups.*

*Statistically significant correlations (p < 0.05) are shown in bold font.*

The prevalence of CRISPR-Cas systems among *Klebsiella* strains was very diverse using CRISPERFinder analysis (Ostria-Hernandez et al., 2015; Shen et al., 2017). CRISPERFinder with the Vmatch program to browse the maximal repeats to get possible CRISPR localization, following by direct consensus DR selection according to candidate occurrences and a score computation. After DR and spacer size check, the tandem repeats are eliminated using ClustalW for aligning spacers (Grissa et al., 2007). The presence of *cas* genes was not verified by CRISPERFinder. Previous report indicated that Cas1-Cas2 complex formation played a critical role in mediating spacer acquisition during CRISPR-Cas adaptive immunity (Nunez et al., 2014). Therefore, we suggested that CRISPERFinder is useful for screening for CRISPR-Cas system, however, the presence of Cas genes/proteins should be validated.

The phylogenetic trees showed two types of Cas1 or Cas3 proteins (Supplementary Figure S1). Twenty-three strains (23/40, 57.5%) had Cas1 and Cas3 alleles (type A) corresponding to CRISPR-Cas type I-E, while the remaining strains (17/40, 42.5%) had Cas1 and Cas3 alleles (type B) consistent with CRISPR-Cas I-E^∗^ (**Table 1** and Supplementary Figure S1). Moreover, the identity of protein sequences between type A Cas1 and type B Cas1 was 35%, and the identity of protein sequences between type A Cas3 and type B Cas3 was 33%. Therefore, the CRISPR type-specific primers for *cas1* and *cas3* genes were designed for determining the distribution of CRISPR-Cas systems in clinical *K. pneumoniae* isolates (Supplementary Table S1).

Shen et al. (2017) reported that type I-E and subtype I-E^∗^ CRISPR-Cas systems were detected in *K. pneumoniae* with different locations in the chromosome. Type I-E CRISPR-Cas system was located in the *iap*-*cysH* region, and subtype I-E^∗^ CRISPR-Cas system was located in the ABC transport system-glyoxalase region (Shen et al., 2017). Here, we revealed that type I-E and subtype I-E^∗^ CRISPR-Cas systems in *K. pneumoniae* could be typed based on the amino sequences of Cas1 and Cas3. However, the function/activity of Cas proteins in different CRISPR-Cas type systems in *K. pneumoniae* remains to be determined.

**FIGURE 1:**
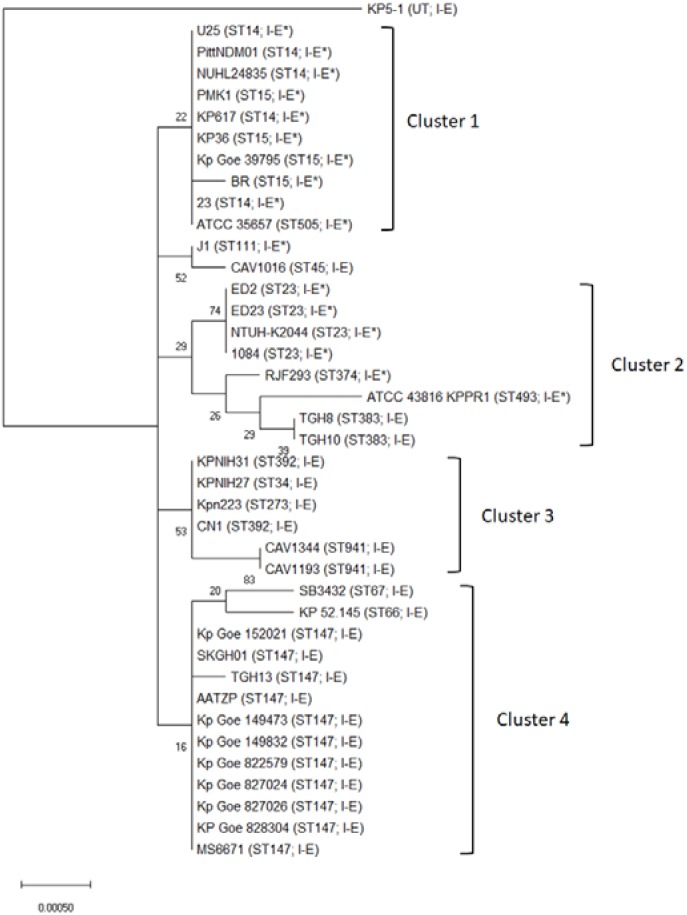
Phylogenetic tree of the concatenated MLST sequences of 40 *Klebsiella pneumoniae* isolates. The phylogenetic tree was based on the aligned nucleotide sequences of concatenated MLST sequences of 40 *K. pneumoniae* isolates by using the Maximum Likelihood method. The percentage of trees in which the associated taxa clustered together is shown next to the branches. The tree is drawn to scale, with branch lengths measured in the number of substitutions per site. Sequence types and CRISPR-Cas subtypes appear in brackets. UT, untypable.

Type I-E CRISPR-Cas was found in ST34, 45, 66, 67, 147, 273, 383, 392, and 941, while subtype I-E^∗^ CRISPR-Cas was present in ST14, 15, 23, 28, 111, 374, 493, and 505 (Supplementary Table S2). The distribution of CRISPR-Cas systems was strongly associated with certain STs (Supplementary Table S2, *p* < 0.001). To better understand any MLST relationship with the CRISPR-Cas types across the *K. pneumoniae*, phylogenetic tree of 40 published *K. pneumoniae* genomes based on aligned nucleotide sequences of concatenated MLST sequences is shown in **Figure 1**. Thirty-seven isolates were assigned to four MLST clusters (**Figure 1**). Nearly all cluster 1 and cluster 2 isolates contained type I-E^∗^ CRISPR-Cas system (except cluster 2 isolates, TGH8 and TGH10). All cluster 3 (*n* = 6) and cluster 4 (*n* = 13) isolates contained type I-E CRISPR-Cas system (**Figure 1**). Three unclustered isolates, KP5-1, J1, and CAV1016, contained type I-E, type I-E^∗^, and type I-E CRISPR-Cas system, respectively (**Figure 1**). Moreover, all ST14 (*n* = 5), 15 (*n* = 4), and 23 (*n* = 4) isolates contained type I-E^∗^ CRISPR-Cas system. In contrast, all ST147 (*n* = 11), 383 (*n* = 2), 392 (*n* = 2), and 941 (*n* = 2) isolates contained type I-E CRISPR-Cas system. These results showed that there was a significant MLST association with the distribution of the type I-E and I-E^∗^ CRISPR-Cas systems across *K. pneumoniae* (**Figure 1**).

### CRISPR-Cas Systems in Clinical *K. pneumoniae* Isolates

Overall, 30.7% of clinical *K. pneumoniae* isolates had CRISPR-Cas systems in 176 *K. pneumoniae* strains isolated from patients with BSI (*n* = 87) or UTI (*n* = 89) in Taiwan. There were no significant differences in the frequency of presence of CRISPR-Cas systems in *K. pneumoniae* between BSI (34.5%, 30/87) or UTI (27.0%, 24/89) strains (*p* > 0.05). However, more type I-E CRISPR-Cas systems were found in UTI isolates and more subtype I-E^∗^ CRISPR-Cas systems were found in BSI isolates (*p* = 0.042). The type I-E CRISPR-Cas systems had 5 (5.7%) and 10 (11.2%) isolates in BSI and UTI isolates, respectively. The subtype I-E^∗^ CRISPR-Cas systems had 25 (28.7%) and 14 (15.7%) in BSI and UTI isolates, respectively.

**FIGURE 2:**
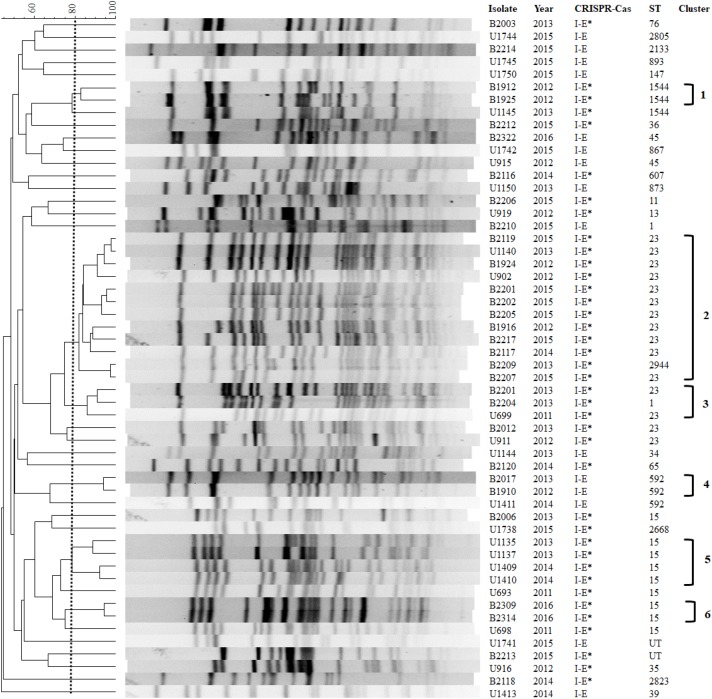
Pulsed-field gel electrophoresis (PFGE) patterns, CRISPR-Cas systems, and STs among 54 clinical *K. pneumoniae* isolates. The first letter of the name of isolates is based on the origins of isolate. U, urinary tract infection; B, bloodstream infection; UT, untypable.

We further determined whether the PFGE typing was associated with the presence of CRISPR-Cas subtypes (**Figure 2**). The PFGE patterns of 54 isolates showed high diversity, and only 25 isolates (46.3%) were assigned to six clusters based on >80% pattern similarity (**Figure 2**). All clusters 1 (*n* = 2), 2 (*n* = 12), 3 (*n* = 3), 5 (*n* = 4), and 6 (*n* = 2) isolates contained type I-E^∗^ CRISPR-Cas system. Two cluster 4 isolates contained type I-E CRISPR-Cas system. The PFGE results suggest that the distribution of CRISPR-Cas subtypes was associated with PFGE typing (**Figure 2**).

We next performed MLST analysis to understand the association of STs with the distribution of CRISPR-Cas types among clinical isolates (**Figure 2**). The results showed that 52 isolates studied comprised 24 different MLST types (isolates B2213 and U1741 were untypable). The most prevalent sequence type was ST23 (11 BSI isolates and 4 UTI isolates), followed by ST15 (3 BSI isolates and 6 UTI isolates). Type I-E CRISPR-Cas was found in ST147, 592, 867, and 873, while subtype I-E^∗^ CRISPR-Cas was present in ST1, 11, 13, 15, 23, 35, 76, 607, 1544, and 2668 (**Figure 2**). The results were consistent with the distribution of CRISPR-Cas systems among published genomes which revealed that ST34, ST45, and ST147 strains contained Type I-E CRISPR-Cas and ST15 and ST23 strains contained subtype I-E^∗^ CRISPR-Cas (**Figure 1**). Interestingly, no CRISPR-Cas system was identified in eight ST11 published genomes but isolate B2206 (ST11) contained subtype I-E^∗^ CRISPR-Cas (**Figure 2** and Supplementary Table S2). Karah et al. (2015) reported that a vertical transmission of the CRISPR-Cas subtype I-Fb in a global collection of 76 *Acinetobacter baumannii* isolates with occasional events of horizontal transfer have increased the diversification and facilitated further dissemination of subtype I-Fb. Taken together, these results raised the possibility of horizontal transfer of CRISPR-Cas systems among *K. pneumoniae* isolates. Moreover, CRISPR-based genotyping approach has been expanded to the analysis of populations and dissemination routes of particular isolates, with the ability to truly assess genetic diversity even within relatively clonal set of bacteria (Karah et al., 2015; Barrangou and Dudley, 2016). Therefore, it is interesting to determine the efficacy of CRISPR typing in epidemiological surveillance and outbreak investigation of *K. pneumoniae* in the future.

Our results revealed that PFGE-clusters 1, 4, 5, and 6, were composed of single sequence type (**Figure 2**). Cluster 2 was composed of ST23 and ST2944 isolates, and cluster 3 was composed of ST1 and ST23 isolates (**Figure 2**). However, 1 ST1544 (U1145), 2 ST23 (B2012 and U911), 1 ST592 (U1411), and 3 ST15 isolates (B2006, U693, and U698), were not included in six PFGE-clusters. These results suggest that MLST had greater discriminatory ability than PFGE to detect the presence of CRISPR-Cas types.

### Subtype I-E^∗^ CRISPR-Cas System Is Associated With Antibiotic Susceptibility and Lower Phage and Plasmid Counts

Isolates having the subtype I-E^∗^ CRISPR-Cas system were more susceptible to ampicillin-sulbactam (76.9%), cefazolin (79.5%), cefuroxime (79.5%), cefotaxime (82.1%), gentamicin (87.2%), and sulfamethoxazole-trimethoprim (76.9%), compared to CRISPR-negative and type I-E CRISPR-Cas isolates (**Table 2**). The antibiotic susceptibilities to ampicillin-sulbactam (*p* = 0.033) and gentamicin (*p* = 0.032) were statistically different between three CRISPR-Cas groups (**Table 2**). Moreover, isolates having the subtype I-E^∗^ CRISPR-Cas system were more susceptible to ampicillin-sulbactam (*p* = 0.009), cefazolin (*p* = 0.016), cefuroxime (*p* = 0.039), and gentamicin (*p* = 0.012), compared to CRISPR-negative isolates (**Table 2**). In contrast, isolates having the type I-E CRISPR-Cas system showed no significant difference of nine antibiotic susceptibilities compared to CRISPR-negative isolates.

**Table 2:** The association of nine antibiotic susceptibilities with CRISPR-Cas systems in clinical *K. pneumoniae* isolates.

	CRISPR-Cas systems			
	Type I-E (*n* = 15)	Type I-E^∗^ (*n* = 39)	Absent (*n* = 122)	*p*-Value
Number of susceptible isolate (%)				
Amikacin	15 (100)	37 (94.9)	116 (95.1)	0.676
Ampicillin-sulbactam	9 (60.0)	30 (76.9)	65 (53.3)	**0.033**
Cefazolin	9 (60.0)	31 (79.5)	71 (58.2)	0.055
Cefuroxime	9 (60.0)	31 (79.5)	75 (61.5)	0.109
Cefotaxime	11 (73.3)	32 (82.1)	83 (68.0)	0.237
Ertapenem	15 (100)	37 (94.9)	112 (91.8)	0.450
Gentamicin	12 (80.0)	34 (87.2)	81 (66.4)	**0.032**
Levofloxacin	13 (86.7)	32 (82.1)	86 (70.5)	0.186
Sulfamethoxazole-trimethoprim	9 (60.0)	30 (76.9)	74 (60.7)	0.171

*Statistically significant correlations (*p* < 0.05) are shown in bold font.*

Our data showed that strains having subtype I-E^∗^ CRISPR-Cas systems were more susceptible to antibiotics (**Table 2**). The results raised the possibility that the subtype I-E^∗^ CRISPR-Cas system efficiently limits the acquisition of acquired antibiotic resistance genes and external DNA fragments. Therefore, the association of chromosome size, numbers of plasmid and phage with the presence of CRISPR-Cas systems in published *K. pneumoniae* genomes were analyzed. The results showed that the average chromosome size between three groups were similar (*p* > 0.05) (**Table 1**). Importantly, strains that had subtype I-E^∗^ CRISPR-Cas systems had significantly decreased numbers of plasmid and phage counts compared with CRISPR type I-E strains or CRISPR-negative strains (**Table 1**). However, the numbers of spacer hit plasmids in subtype I-E^∗^ CRISPR-strains were similar to type I-E CRISPR strains (*p* = 0.318) (**Table 1** and Supplementary Table S3). We next analyzed the spacer sequences of subtype I-E^∗^ CRISPR-Cas systems in 17 published genomes. The results showed that the spacers of subtype I-E^∗^ CRISPR-Cas systems targeted sequences match genes encoded phage-related proteins (including phage integrase, tail, portal, and holin family proteins), conjugal transfer proteins, and IS3 family transposase. These results suggested that subtype I-E^∗^ CRISPR-Cas system efficiently limited the acquisition of external DNA fragments and phage infections.

We next examined the distribution of acquired antibiotic resistance genes among 97 published genomes (**Table 1**). The results showed a low frequency of aminoglycoside and MLS resistance genes among CRISPR subtype I-E^∗^ and type I-E strains, respectively (**Table 1**). Moreover, strains containing CRISPR subtype I-E^∗^ had lower numbers of β-lactam (*p* = 0.026) and fluoroquinolone resistance genes (*p* = 0.037) compared with CRISPR-negative and type I-E strains, respectively (**Table 1**). However, no subtype I-E^∗^ CRISPR-Cas-targeting spacer sequences complementary to any known antibiotic resistance genes were identified. A small number of spacer target sequences did match known and previously studied antibiotic resistance-encoding plasmids. Among these spacers, spacer sequences CCGGCATCCGTCAGCTCGACGGCCAGCTGCAG (and its complementary sequence) and CCGCCGTTTAATCGCGGTGATGATATCCGGCA (and its complementary sequence) were found in 13 (76.5%) and 10 (58.8%) subtype I-E^∗^ CRISPR-Cas systems, respectively (Supplementary Table S3). Moreover, nucleotide BLAST results showed that these two spacers matched short regions within conjugatable IncFIB-, IncFII, IncN-, and IncQ1-type plasmids carrying several antibiotic resistance genes.

ST11 and ST258, belonged to clonal complex 258 (CC258), are well-established to comprise the largest MDR *K. pneumoniae* clonal group worldwide (Pitout et al., 2015; Lee et al., 2016). ST258 is the predominant clone associated with the spread of KPC-producing *K. pneumoniae* observed in European countries and the United States (Bowers et al., 2015), and ST11 is the prevalent KPC-producing clone in China and Taiwan (Tseng et al., 2015; Dong et al., 2018). Here, we revealed no CRISPR-Cas system was identified in ST11 (*n* = 8) and ST258 (*n* = 16) published genomes (Supplementary Table S2). Moreover, isolate B2206 (ST11) contained subtype I-E^∗^ CRISPR-Cas showed susceptibility to amikacin, ertapenem, gentamicin, and levofloxacin. Taken together, these results suggest the direct association between absence of CRISPR-Cas system and MDR-phenotype of CC258 *K. pneumoniae* isolates.

CRISPR-Cas systems are associated with antibiotic susceptibility in *S. pyogenes*, *Pseudomonas aeruginosa* and *E. coli* (Zheng et al., 2014; van Belkum et al., 2015; Aydin et al., 2017). Aydin et al. (2017) reported that five Type I-F spacers matched conserved regions within IncFII-, IncFIB-, and IncI1-type plasmids which are associated with the spread of antibiotic resistance genes. Taken together, these results suggest that CRISPR-Cas systems, especially subtype I-E^∗^, potentially interfere with the acquisition of antibiotic resistance plasmids, maintaining susceptibility in *K. pneumoniae* isolates. However, the sequences of spacers among clinical *K. pneumoniae* isolates having subtype I-E^∗^ CRISPR systems remain to be determined to clarify their roles in limiting external DNA acquisition.

In summary, we first characterized the CRISPR-Cas systems in clinical *K. pneumoniae* isolates. The results showed that the distribution of CRISPR-Cas subtypes was directly associated with PFGE typing. Importantly, isolates that had subtype I-E^∗^ CRISPR-Cas system were more susceptible to certain antibiotics, compared to CRISPR-negative isolates, and the results were consistent with our observation from published genomes.

## Author Contributions

H-YL and C-YK designed the study and performed analyses. W-HL, P-XZ, J-JY, M-CW, C-HT, C-CT, and J-JW participated, coordinated, and supervised the study. H-YL, C-YK, and J-JW wrote the manuscript. All authors approved the final manuscript.

## Conflict of Interest Statement

The authors declare that the research was conducted in the absence of any commercial or financial relationships that could be construed as a potential conflict of interest.

## Footnotes

1 https://img.jgi.doe.gov/

2 http://crispr.i2bc.paris-saclay.fr/Server/

3 https://blast.ncbi.nlm.nih.gov/

4 http://bigsdb.pasteur.fr/klebsiella/

5 http://phast.wishartlab.com/

6 https://cge.cbs.dtu.dk/services/ResFinder/
