# Supplementary material for: Characterization of CRISPR-Cas Systems in Clinical Klebsiella pneumoniae Isolates Uncovers Its Potential Association With Antibiotic Susceptibility
Source: Front Microbiol. 2018 Jul 16;9:1595. doi: 10.3389/fmicb.2018.01595 (PMC6054925; doi:10.3389/fmicb.2018.01595)
Supplement: Supplementary file 1 [file Table_1.doc]

**Material and Methods**

**ST type determination of *K. pneumoniae* published genomes**

A total of 97 complete chromosome sequences of *K. pneumoniae* were retrieved from the Integrated Microbial Genomes & Microbiomes (IMG/M) genome database (https://img.jgi.doe.gov/). Sequence types (STs) were determined by comparing the whole-genome sequences against the *K. pneumoniae* Pub MLST database (http://bigsdb.pasteur.fr/klebsiella/).

**Spacer analysis and plasmid hits**

CRISPRFinder was used to identify the spacers of CRISPR-Cas system in 40 CRISPR-positive genomes (http://crispr.i2bc.paris-saclay.fr/Server/). BLASTn was used to determine the plasmid hits of spacer sequences (https://blast.ncbi.nlm.nih.gov/).

**Phylogenetic trees building**

Neighbor-Joining phylogenetic trees were constructed using the CLC sequence viewer alignments of the Cas1 or Cas3 protein sequences.

**Table S1. Oligonucleotide primers used in this study**

| Primer | Sequence (5’-3’) | Annealing Tm | Product (bp) |
| --- | --- | --- | --- |
| Cas1 type A | CGAAACGCTACGGTGTGAAA | 60 | 341 |
|  | CGGAATCAATTTGCCTGTCA |  |  |
| Cas1 type B | CGGCTGGAAATTGATGACAG | 60 | 309 |
|  | ATCCGGAAAGCGTTTAGCAA |  |  |
| Cas3 type A | GCCGCTTTGCCTTTTATGAC | 60 | 362 |
|  | GCCGTGATCGGGATAGACAT |  |  |
| Cas3 type B | AGTCTGGAGGTCTGGCAAGC | 60 | 407 |
|  | CGCACCAGCTGCTGATTAAG |  |  |

**Table S2. The distribution of CRISPR-Cas systems and ST types among 97 *K. pneumoniae* genomes**

|  | CRISPR-Cas systems | | |
| --- | --- | --- | --- |
|  | Presence (n = 40) | | Absence (n = 57) |
|  | Type I-E (n = 23) | Type I-E* (n = 17) |
| **ST types** |  |  |  |
| 11 | 0 | 0 | 8 |
| 14 | 0 | 5 | 0 |
| 15 | 0 | 4 | 0 |
| 16 | 0 | 0 | 2 |
| 23 | 0 | 4 | 1 |
| 34 | 1 | 0 | 1 |
| 37 | 0 | 0 | 1 |
| 38 | 0 | 0 | 1 |
| 45 | 1 | 0 | 0 |
| 65 | 0 | 0 | 1 |
| 66 | 1 | 0 | 0 |
| 67 | 1 | 0 | 0 |
| 86 | 0 | 0 | 2 |
| 101 | 0 | 0 | 3 |
| 111 | 0 | 1 | 0 |
| 146 | 0 | 0 | 1 |
| 147 | 11 | 0 | 0 |
| 244 | 0 | 0 | 1 |
| 258 | 0 | 0 | 16 |
| 273 | 1 | 0 | 0 |
| 278 | 0 | 0 | 2 |
| 340 | 0 | 0 | 3 |
| 374 | 0 | 1 | 0 |
| 375 | 0 | 0 | 1 |
| 383 | 2 | 0 | 0 |
| 392 | 2 | 0 | 0 |
| 395 | 0 | 0 | 2 |
| 442 | 0 | 0 | 1 |
| 493 | 0 | 1 | 0 |
| 505 | 0 | 1 | 0 |
| 512 | 0 | 0 | 1 |
| 514 | 0 | 0 | 1 |
| 941 | 2 | 0 | 0 |
| 1518 | 0 | 0 | 1 |
| 1536 | 0 | 0 | 2 |
| 2549 | 0 | 0 | 1 |
| 2624 | 0 | 0 | 1 |
| Untypable | 0 | 0 | 4 |

**Table S3. Type of CRISPR-Cas systems, number of spacers, and number of plasmid hits by spacer sequences among 40 CRISPR-Cas positive genomes**

|  |  | CRISPR-Cas systems | | | |
| --- | --- | --- | --- | --- | --- |
| Strain | Accession number | Type | Spacer (n) | Spacer | Plasmid hits (n) |
| NTUH-K2044 | AP006725.1 | I-E* | 25 | - | - |
| 1084 | CP003785.1 | I-E* | 22 | CCTGCAGCTGGCCGTCGAGCTGACGGATGCCGG | 63 |
|  |  |  |  | TACACCCAGCTCTTTGACGCAAAGGCTCAGGAG | 2 |
|  |  |  |  | CTGGTTGACGTATGCCGTGATGCTGCTGGTAGG | 2 |
| SB3432 | FO203501.1 | I-E | 2 | GTCTTCCCTGTTTGCTGCCTGCTGTCTGTCTG | 84 |
| KPNIH27 | CP007731.1 | I-E | 39 | TTTGGTATTTGTGCTGATTACCCGTTTCAGTA | 5 |
| PittNDM01 | CP006798.1 | I-E* | 21 | TGCCGGATATCATCACCGCGATTAAACGGCGGA | 81 |
|  |  |  |  | CCGGCATCCGTCAGCTCGACGGCCAGCTGCAG | 63 |
| ATCC 43816 KPPR1 | CP009208.1 | I-E* | 17 | CGAAGATGTTCATCCTGACGATAGCAAGAAAAT | 2 |
|  |  |  |  | CCGCCGTTTAATCGCGGTGATGATATCCGGCA | 81 |
| KPNIH31 | CP009876.1 | I-E | 39 | GTCTTCCCTGTTTGCTGCCTGCTGTCTGTCTG | 82 |
|  |  |  |  | GGGGACCTGCTGAACCTGCCCCCTGGTATTAA | 74 |
|  |  |  |  | GAGGTGAGTTCAGTCGAAACGCCCGGTTATCG | 84 |
|  |  |  |  | GCGCTCTTCGTCGTTGCGGCGGGTGCCTGCTC | 89 |
| KP 52.145 | FO834906 | I-E | 18 | - | - |
| TGH8 | CP012743.1 | I-E | 24 | TGCTTTATGGCAAATAAGAGAGGATATAACCA | 2 |
| KP617 | CP012753.1 | I-E* | 21 | CTGCAGCTGGCCGTCGAGCTGACGGATGCCGG | 63 |
|  |  |  |  | TCCGCCGTTTAATCGCGGTGATGATATCCGGCA | 81 |
| SKGH01 | CP015500.1 | I-E | 43 | GAGCAGGCACCCGCCGCAACGACGAAGAGCGC | 89 |
|  |  |  |  | CGATAACCGGGCGTTTCGACTGAACTCACCTC | 84 |
|  |  |  |  | TTAATACCAGGGGGCAGGTTCAGCAGGTCCCC | 74 |
|  |  |  |  | CAGACAGACAGCAGGCAGCAAACAGGGAAGAC | 84 |
| NUHL24835 | NZ_CP014004 | I-E* | 24 | CTGCAGCTGGCCGTCGAGCTGACGGATGCCGG | 63 |
|  |  |  |  | TCCGCCGTTTAATCGCGGTGATGATATCCGGCA | 81 |
| AATZP | CP014755.1 | I-E | 43 | GTCTTCCCTGTTTGCTGCCTGCTGTCTGTCTG | 82 |
|  |  |  |  | GGGGACCTGCTGAACCTGCCCCCTGGTATTAA | 74 |
|  |  |  |  | GAGGTGAGTTCAGTCGAAACGCCCGGTTATCG | 84 |
|  |  |  |  | GCGCTCTTCGTCGTTGCGGCGGGTGCCTGCTC | 89 |
| J1 | CP013711.1 | I-E* | 22 | CCGGCATCCGTCAGCTCGACGACCAGCTGCAGG | 62 |
|  |  |  |  | CAGCGCAGAGCAGGCCAAGGGGCGCGATGACCA | 1 |
| U25 | CP012043.1 | I-E* | 21 | TGCCGGATATCATCACCGCGATTAAACGGCGGA | 81 |
|  |  |  |  | TCATGCTGCTCTATCTCCAAATCGCGCCTTCCA | 1 |
|  |  |  |  | TCGCCGTCGAAGTGCTGCGCGATAGGGATGATA | 2 |
|  |  |  |  | CCGGCATCCGTCAGCTCGACGGCCAGCTGCAG | 63 |
|  |  |  |  | CACGTGATCGCCCTGGCGCGGACGCCGGGAGGT | 1 |
| MS6671 | LN824133.1 | I-E | 43 | GTCTTCCCTGTTTGCTGCCTGCTGTCTGTCTG | 82 |
|  |  |  |  | GGGGACCTGCTGAACCTGCCCCCTGGTATTAA | 74 |
|  |  |  |  | GAGGTGAGTTCAGTCGAAACGCCCGGTTATCG | 84 |
|  |  |  |  | GCGCTCTTCGTCGTTGCGGCGGGTGCCTGCTC | 89 |
| TGH10 | CP012744.1 | I-E | 24 | - | - |
| PMK1 | CP008929.1 | I-E* | 17 | CCGGCATCCGTCAGCTCGACGGCCAGCTGCAG | 63 |
|  |  |  |  | TGCCGGATATCATCACCGCGATTAAACGGCGG | 81 |
|  |  |  |  | GCTAACCAGTGGATAGAGCACTATGTGACGAC | 6 |
|  |  |  |  | GCCACCGGCGGCGCCGAGATCGGGCCATGCGA | 6 |
| 23 | CP016926.1 | I-E* | 20 | ACCTCCCGGCGTCCGCGCCAGGGCGATCACGTG | 1 |
|  |  |  |  | CTGCAGCTGGCCGTCGAGCTGACGGATGCCGG | 63 |
|  |  |  |  | ATCATCCCTATCGCGCAGCACTTCGACGGCGA | 2 |
|  |  |  |  | CCGCCGTTTAATCGCGGTGATGATATCCGGCA | 81 |
| CAV1193 | CP013322.1 | I-E | 45 | TTAATACCAGGGGGCAGGTTCAGCAGGTCCCC | 75 |
|  |  |  |  | CAGACAGACAGCAGGCAGCAAACAGGGAAGAC | 82 |
| CAV1016 | CP017934.1 | I-E | 64 | GTGGTTTGTTACCGTGTTGTGTGGCAAAAAGC | 47 |
|  |  |  |  | GAACGGAGGAATATAAGAACAAAAGCCCGCAG | 46 |
|  |  |  |  | CGATAACCGGGCGTTTCGACTGAACTCACCTC | 83 |
|  |  |  |  | TTAATACCAGGGGGCAGGTTCAGCAGGTCCCC | 75 |
|  |  |  |  | TCGTCTGAGTTCCGGCTTACGCCGTGCCGACA | 76 |
|  |  |  |  | CAGACAGACAGCAGGCAGCAAACAGGGAAGAC | 82 |
| Kp_Goe_149832 | CP018695.1 | I-E | 35 | GAGCAGGCACCCGCCGCAACGACGAAGAGCGC | 92 |
|  |  |  |  | CGATAACCGGGCGTTTCGACTGAACTCACCTC | 83 |
|  |  |  |  | TTAATACCAGGGGGCAGGTTCAGCAGGTCCCC | 75 |
|  |  |  |  | CAGACAGACAGCAGGCAGCAAACAGGGAAGAC | 82 |
| CAV1344 | CP011624.1 | I-E | 45 | TTAATACCAGGGGGCAGGTTCAGCAGGTCCCC | 75 |
|  |  |  |  | CAGACAGACAGCAGGCAGCAAACAGGGAAGAC | 82 |
| Kp_Goe_152021 | CP018713.1 | I-E | 35 | GAGCAGGCACCCGCCGCAACGACGAAGAGCGC | 92 |
|  |  |  |  | CGATAACCGGGCGTTTCGACTGAACTCACCTC | 83 |
|  |  |  |  | TTAATACCAGGGGGCAGGTTCAGCAGGTCCCC | 75 |
|  |  |  |  | CAGACAGACAGCAGGCAGCAAACAGGGAAGAC | 82 |
| Kp_Goe_149473 | CP018686.1 | I-E | 35 | GAGCAGGCACCCGCCGCAACGACGAAGAGCGC | 92 |
|  |  |  |  | CGATAACCGGGCGTTTCGACTGAACTCACCTC | 83 |
|  |  |  |  | TTAATACCAGGGGGCAGGTTCAGCAGGTCCCC | 75 |
|  |  |  |  | CAGACAGACAGCAGGCAGCAAACAGGGAAGAC | 82 |
| KP36 | CP017385.1 | I-E* | 20 | TGCCGGATATCATCACCGCGATTAAACGGCGG | 81 |
|  |  |  |  | GCTAACCAGTGGATAGAGCACTATGTGACGAC | 6 |
|  |  |  |  | CCGGCATCCGTCAGCTCGACGGCCAGCTGCAG | 63 |
|  |  |  |  | CACGTGATCGCCCTGGCGCGGACGCCGGGAGGT | 1 |
| ED2 | CP016813.1 | I-E* | 19 | CCTGCAGCTGGCCGTCGAGCTGACGGATGCCGG | 63 |
|  |  |  |  | ACACCCAGCTCTTTGACGCAAAGGCTCAGGAG | 2 |
|  |  |  |  | TGGTTGACGTATGCCGTGATGCTGCTGGTAGG | 2 |
|  |  |  |  | GCGGGTGGATGACAATAACGCCTGGCGCGCCG | 1 |
| CN1 | CP015382.1 | I-E | 39 | GAGCAGGCACCCGCCGCAACGACGAAGAGCGC | 92 |
|  |  |  |  | CGATAACCGGGCGTTTCGACTGAACTCACCTC | 83 |
|  |  |  |  | TTAATACCAGGGGGCAGGTTCAGCAGGTCCCC | 75 |
|  |  |  |  | CAGACAGACAGCAGGCAGCAAACAGGGAAGAC | 82 |
| KP_Goe_828304 | CP018719.1 | I-E | 35 | GAGCAGGCACCCGCCGCAACGACGAAGAGCGC | 92 |
|  |  |  |  | CGATAACCGGGCGTTTCGACTGAACTCACCTC | 83 |
|  |  |  |  | TTAATACCAGGGGGCAGGTTCAGCAGGTCCCC | 75 |
|  |  |  |  | CAGACAGACAGCAGGCAGCAAACAGGGAAGAC | 82 |
| ATCC 35657 | CP015134.1 | I-E* | 35 | AGGTCCAGTTCGTGCTGCCGGATAACGCAGCCG | 1 |
|  |  |  |  | TTGACCTTGCCGTTTTCCGTGAGCGCCAGTCGA | 2 |
|  |  |  |  | CAACATTTGCCACCAGCCAGCCGCGAATACTGG | 1 |
|  |  |  |  | TACAGCTCTTTCAGGGGAACGATAAACGTCCGG | 1 |
|  |  |  |  | CCGGCATCCGTCAGCTCGACGGCCAGCTGCAGG | 63 |
|  |  |  |  | ACCCGCAGCGCTATCCCGCCGCAGATGATGGAA | 3 |
|  |  |  |  | TCGCCCGCCGACTCCAGCGACTGAATGAGCTCG | 4 |
| TGH13 | CP012745.1 | I-E | 41 | GAGCAGGCACCCGCCGCAACGACGAAGAGCGC | 92 |
|  |  |  |  | CGATAACCGGGCGTTTCGACTGAACTCACCTC | 83 |
|  |  |  |  | TTAATACCAGGGGGCAGGTTCAGCAGGTCCCC | 75 |
|  |  |  |  | CAGACAGACAGCAGGCAGCAAACAGGGAAGAC | 82 |
| KP5-1 | CP012426.1 | I-E | 43 | GAGCAGGCACCCGCCGCAACGACGAAGAGCGC | 92 |
|  |  |  |  | CGATAACCGGGCGTTTCGACTGAACTCACCTC | 83 |
|  |  |  |  | TTAATACCAGGGGGCAGGTTCAGCAGGTCCCC | 75 |
|  |  |  |  | CAGACAGACAGCAGGCAGCAAACAGGGAAGAC | 82 |
| RJF293 | CP014008.1 | I-E* | 13 | AGAACGAATGCCCGCGCTGGTACGGCGCGTCGTGGATTCCA | 2 |
|  |  |  |  | AGTAAAGCCCCGGCGCTAACCGGGGCGGTGTCG | 24 |
|  |  |  |  | CCGGCATCCGTCAGCTCGACGGCCAGCTGCAG | 63 |
|  |  |  |  | ACCCGCAGCGCTATCCCGCCGCAGATGATGGA | 3 |
| ED23 | CP016814.1 | I-E* | 25 | AGGTCCAGTTCGTGCTGCCGGATAACGCAGCCG | 1 |
|  |  |  |  | CGGCGCGCCAGGCGTTATTGTCATCCACCCGCA | 1 |
|  |  |  |  | GGGCGTGAAGGTTATCGATGGCCACCACCGCTA | 1 |
|  |  |  |  | GTAGTCGGCGCGTCGGTGATAGAGATCGTGGTG | 1 |
| Kp_Goe_827026 | CP018707.1 | I-E | 35 | GAGCAGGCACCCGCCGCAACGACGAAGAGCGC | 92 |
|  |  |  |  | CGATAACCGGGCGTTTCGACTGAACTCACCTC | 83 |
|  |  |  |  | TTAATACCAGGGGGCAGGTTCAGCAGGTCCCC | 75 |
|  |  |  |  | CAGACAGACAGCAGGCAGCAAACAGGGAAGAC | 82 |
| Kpn223 | CP015025.1 | I-E | 43 | GTCTTCCCTGTTTGCTGCCTGCTGTCTGTCTG | 82 |
|  |  |  |  | GGGGACCTGCTGAACCTGCCCCCTGGTATTAA | 75 |
|  |  |  |  | GAGGTGAGTTCAGTCGAAACGCCCGGTTATCG | 83 |
|  |  |  |  | GCGCTCTTCGTCGTTGCGGCGGGTGCCTGCTC | 92 |
| BR | CP015990.1 | I-E* | 20 | TGCCGGATATCATCACCGCGATTAAACGGCGG | 81 |
|  |  |  |  | GCTAACCAGTGGATAGAGCACTATGTGACGAC | 6 |
|  |  |  |  | GCTACTGCATCCACGGCGTACATGCTCAGTGT | 1 |
|  |  |  |  | TGCCGCAGACCGGGCAGCGCGGTATGTCCGTC | 1 |
|  |  |  |  | GCCACCGGCGGCGCCGAGATCGGGCCATGCGA | 6 |
|  |  |  |  | CCGGCATCCGTCAGCTCGACGGCCAGCTGCAG | 63 |
| Kp_Goe_822579 | CP018140.1 | I-E | 35 | GAGCAGGCACCCGCCGCAACGACGAAGAGCGC | 95 |
|  |  |  |  | CGATAACCGGGCGTTTCGACTGAACTCACCTC | 86 |
|  |  |  |  | TTAATACCAGGGGGCAGGTTCAGCAGGTCCCC | 77 |
|  |  |  |  | CAGACAGACAGCAGGCAGCAAACAGGGAAGAC | 84 |
| Kp_Goe_39795 | CP018458.1 | I-E* | 20 | ACCTCCCGGCGTCCGCGCCAGGGCGATCACGTG | 1 |
|  |  |  |  | CTGCAGCTGGCCGTCGAGCTGACGGATGCCGG | 63 |
|  |  |  |  | TCGCATGGCCCGATCTCGGCGCCGCCGGTGGC | 5 |
|  |  |  |  | GACGGACATACCGCGCTGCCCGGTCTGCGGCA | 1 |
|  |  |  |  | ACACTGAGCATGTACGCCGTGGATGCAGTAGC | 1 |
|  |  |  |  | GTCGTCACATAGTGCTCTATCCACTGGTTAGC | 6 |
|  |  |  |  | CCGCCGTTTAATCGCGGTGATGATATCCGGCA | 81 |
| Kp_Goe_827024 | CP018701.1 | I-E | 35 | GAGCAGGCACCCGCCGCAACGACGAAGAGCGC | 95 |
|  |  |  |  | CGATAACCGGGCGTTTCGACTGAACTCACCTC | 86 |
|  |  |  |  | TTAATACCAGGGGGCAGGTTCAGCAGGTCCCC | 77 |

**Figures**

**Fig S1**

**A.**


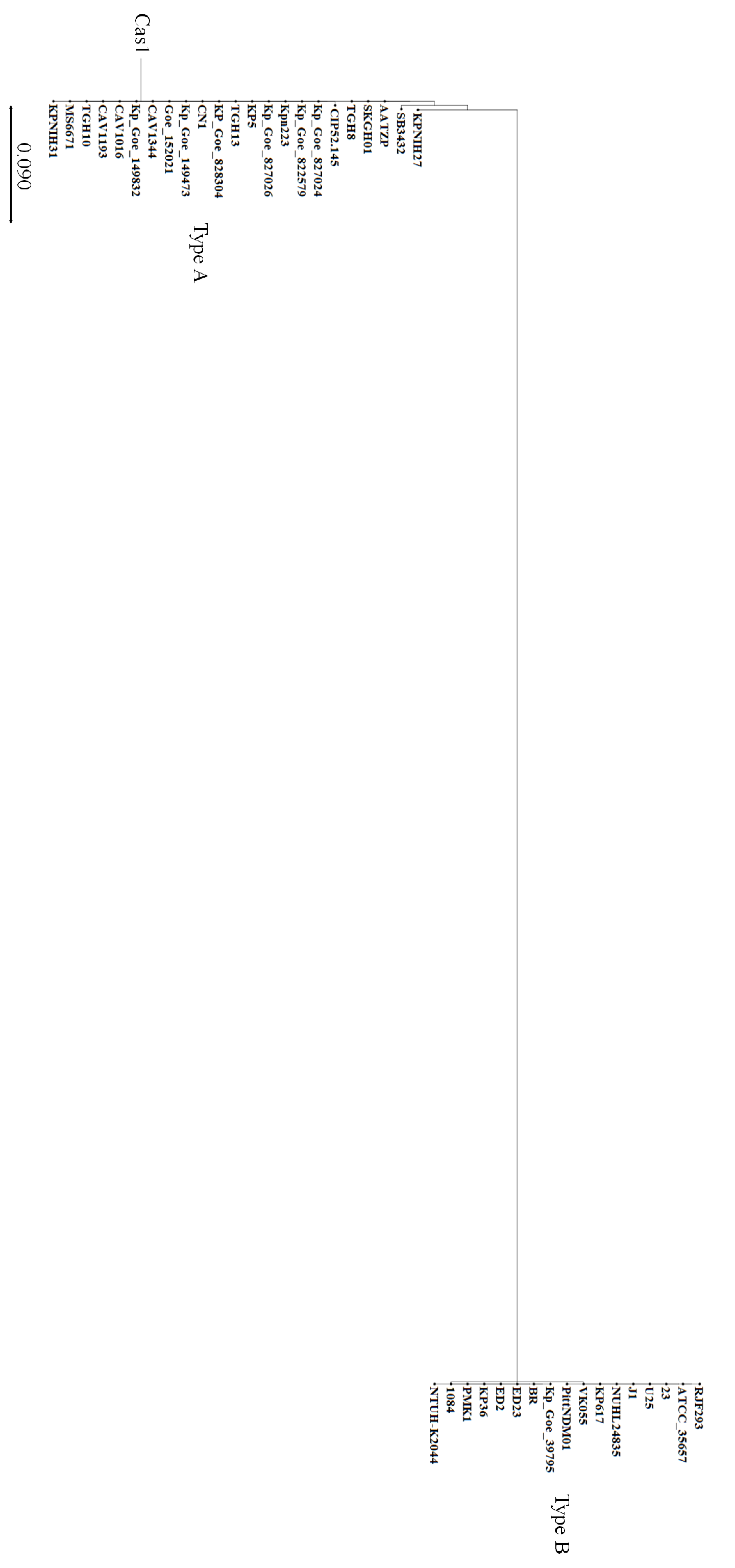


**B.**


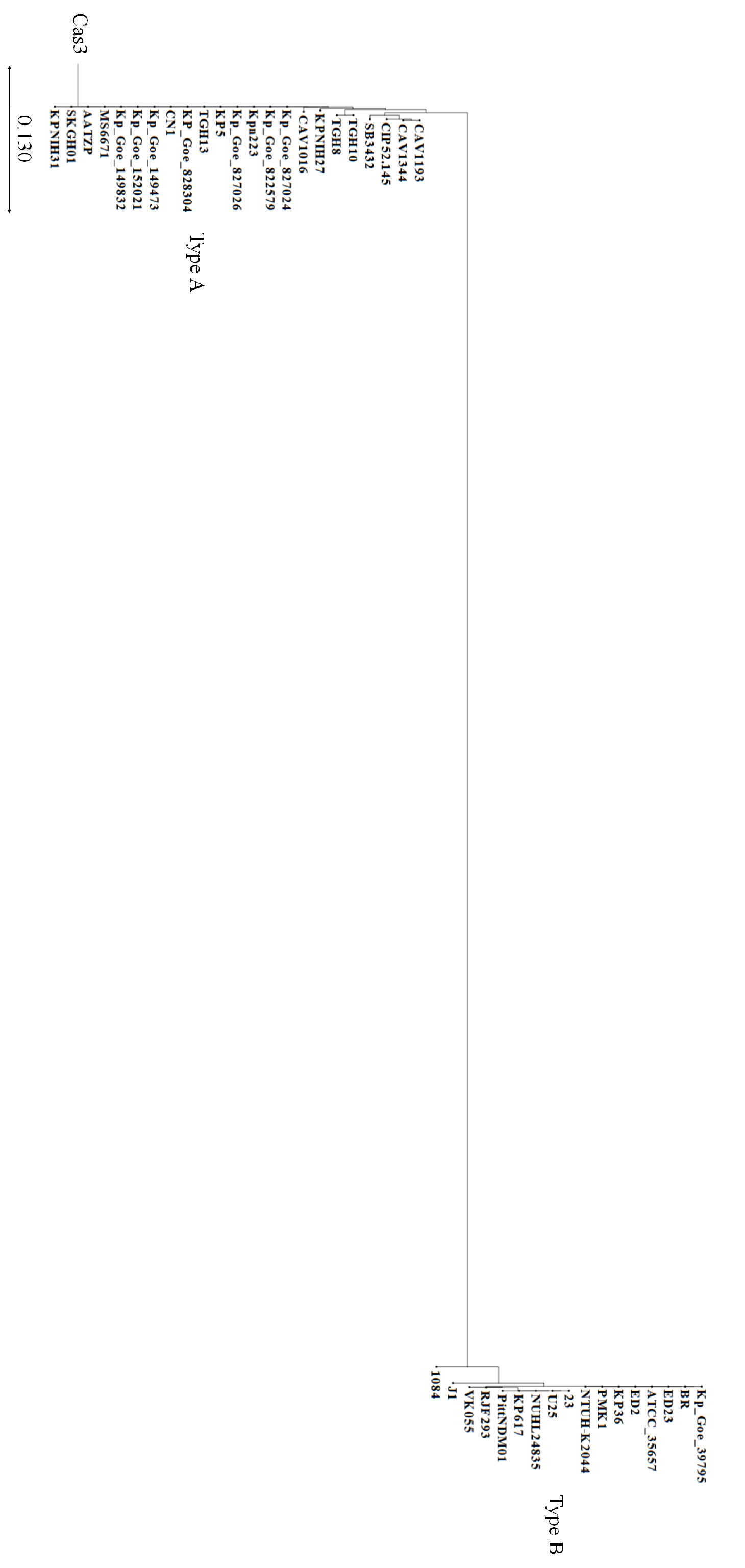


**Figure S1.**  **Phylogenetic trees built on basis of amino acid distances between sequences of Cas1 (A) or Cas3 (B) aligned by Jukes-Cantor distance model**. Numbers of substitutions per site are indicated, along with a scale bar.
